# A Case of Previously Undiagnosed Sturge–Weber Syndrome in an African American Patient due to a Missed Port‐Wine Stain

**DOI:** 10.1155/crnm/1884299

**Published:** 2026-04-10

**Authors:** Lisle Blackbourn, Jayishnu Srinivas, Maher Salem, Usha Janapala

**Affiliations:** ^1^ Department of Neurology, University of Illinois College of Medicine Peoria, Peoria, Illinois, USA, uic.edu; ^2^ Department of Neurology, OSF Illinois Neurological Institute, Peoria, Illinois, USA

## Abstract

Sturge–Weber syndrome (SWS) is a rare, congenital neurocutaneous disorder characterized by the triad of facial capillary malformations, ocular abnormalities, and leptomeningeal angiomatosis which can lead to neurological manifestations such as seizures, intellectual disability, and stroke‐like episodes. This case report describes a 45‐year‐old African American female who presented with status epilepticus and stroke‐like symptoms, ultimately diagnosed with SWS based on characteristic imaging findings, with an apparent previously missed port‐wine stain. This case highlights the diagnostic and management challenges of SWS in adulthood, particularly in African American patients, where the characteristic cutaneous features may be less apparent. The patient’s presentation with status epilepticus and acute infarct underscores the fluctuating or episodic neurologic nature of SWS and the need for prompt diagnosis and management.

## 1. Introduction

Sturge–Weber syndrome (SWS) is a rare, congenital neurocutaneous disorder characterized by the triad of facial capillary malformations, ocular abnormalities, and leptomeningeal angiomatosis which can lead to neurological manifestations such as seizures, intellectual disability, and stroke‐like episodes [1]. First described by William Allen Sturge and Frederick Parkes Weber in the late 19th century, SWS is caused by a somatic mosaic mutation in the guanine nucleotide‐binding protein, alpha subunit q (GNAQ) gene, which leads to abnormal vascular development and endothelial cell proliferation [2]. While the condition is typically diagnosed in infancy or early childhood, cases of late‐onset SWS, particularly in adulthood, are increasingly recognized, though they remain underreported and are often misdiagnosed due to the atypical presentation and the absence of classic cutaneous features [3–5].

This case report describes a 45‐year‐old African American female who presented with status epilepticus and stroke‐like symptoms, ultimately diagnosed with SWS based on characteristic imaging findings, with an apparent previously missed port‐wine stain. This case highlights the diagnostic and management challenges of SWS in adulthood, particularly in African American patients, where the characteristic cutaneous features may be less apparent. The patient’s presentation with status epilepticus and acute infarct underscores the fluctuating or episodic neurologic manifestation nature of SWS and the need for prompt diagnosis and management.

## 2. Case

**FIGURE 1:**
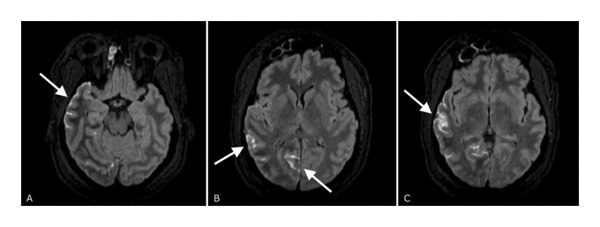
Brain MRI, postcontrast FLAIR sequence, demonstrating gyriform leptomeningeal enhancement over the right temporo‐occipital region (A–C).

A 45‐year‐old African American female with a past medical history of migraines and a remote history of childhood seizures presented from an outside facility for electroclinical status epilepticus. The patient presented to the outside facility for a 5‐day history of headache and fever. She was then found on the floor at the facility, unresponsive, with reports of a “bruise” over her face from the fall. Lactate was elevated at 17.5 mmol/L (0.7–2.0 mmol/L), and an EEG showed focal seizure. Clinically, the patient had left head turn and eye deviation with the episode capture. The patient then went into status epilepticus and was transferred to our facility for further care after intubation. The patient had been started on levetiracetam 1 g twice a day and lacosamide 100 mg twice a day there, which were continued upon arrival. Due to fever and headaches, there was initial concern for meningitis for which she was started on empiric meningitis coverage; however, CSF studies were unremarkable upon infectious testing including HSV. The patient did have a slightly elevated CSF protein of 72 mg/dL (12–60 mg/dL), glucose of 145 mg/dL (40–70 mg/dL), and a total nucleated cell count of 3 per mm^3^ (0–5 per mm^3^). Later, the patient was found to have an *E. coli* urinary tract infection which was treated with ceftriaxone. MRI of the brain with and without contrast showed gyriform leptomeningeal enhancement overlying the right temporal occipital lobes with minimal involvement of the right posterior parietal lobe, a small acute infarct involving the right occipital lobe with T2 FLAIR hyperintensity, and prominent bilateral optic nerve sheath and partially empty sella. The gyriform leptomeningeal enhancement can be seen in Figure 1.

**FIGURE 2:**
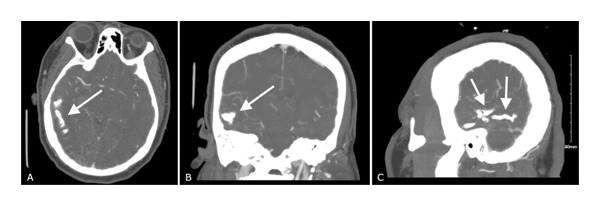
A CT venography of the head demonstrating findings characteristic of Sturge–Weber syndrome of leptomeningeal angiomatosis and gyriform calcifications involving the right posterior cerebral hemisphere as seen in the (A) axial, (B) coronal, and (C) sagittal views.

The patient was started on aspirin 81 mg daily and atorvastatin 20 mg nightly for secondary stroke prevention due to the finding of an acute stroke on MRI. The MRI brain also showed right temporal and occipital parenchymal calcifications, as seen in Figure 1. For this reason, a CT venography of the head was done, which demonstrated findings characteristic of SWS, including leptomeningeal angiomatosis and gyriform calcifications involving the right posterior cerebral hemisphere, as seen in Figure 2. No evidence of venous thrombus was seen.

After extubation and further discussion with the patient, she told us her presumed “bruise” was in fact a birthmark she has always had. Upon follow‐up in the outpatient neurology clinic 2 months later, the patient had no neurologic deficits and was seizure‐free.

## 3. Discussion

Initially, there was concern for an infectious cause of meningoencephalitis given the initial presentation of headaches, fever, and seizures. Further infectious testing later ruled out any infectious causes, and the fever was deemed to be due to a UTI vs. postseizure fever. CSF results also did not point to neoplastic leptomeningeal disease as a cause. Imaging and further clinical history obtained after the patient was extubated led to a diagnosis of SWS.

The pathophysiology of SWS is rooted in a somatic mosaic mutation in the GNAQ gene, which leads to abnormal vascular development and endothelial cell proliferation [2]. Newer research suggests that this mutation causes calcium signaling abnormalities [6]. This mutation results in the hallmark features of SWS, including leptomeningeal angiomatosis, which is often unilateral and involves the occipital and parietal lobes, as seen in this patient [1, 7]. The gyriform calcifications and leptomeningeal enhancement observed on imaging are thought to result from chronic hypometabolism and venous stasis due to the abnormal vasculature [1, 8]. These vascular abnormalities predispose patients to seizures, stroke‐like episodes, and potentially rare progressive neurological decline. In this case, the patient’s history of childhood seizures and migraines aligns with the natural history of SWS, which often includes episodic neurological symptoms [7]. However, the recurrence of seizures in adulthood, culminating in status epilepticus, is less commonly reported and highlights the rare potentially progressive nature of the disease and impact of missing this diagnosis in childhood.

Seizures are the most common neurological manifestation of SWS, occurring in 75%–100% of patients, and typically present in infancy or early childhood [9, 10]. However, cases of SWS diagnosed in adulthood, particularly in patients in their 40s, are rare but not unprecedented. A review of the literature reveals that late‐onset SWS is often underrecognized, as the condition is typically associated with pediatric populations [7]. In adults, SWS may present with new‐onset seizures, stroke‐like episodes, or rare progressive neurological deficits, often leading to misdiagnosis or delayed diagnosis [1, 3, 5]. This patient’s presentation with status epilepticus and focal neurological findings, including left head turn and eye deviation, underscores the importance of considering SWS in the differential diagnosis of adult patients with unexplained seizures, even in the absence of a known history of the disorder.

The imaging findings in this case, including gyriform leptomeningeal enhancement and T2 FLAIR hyperintensity in the right occipital lobe, are characteristic of SWS [1]. The presence of a small acute infarct further supports the diagnosis, as ischemic events are common in SWS due to impaired cerebral perfusion [8, 11, 12]. Due to our patient’s ischemic stroke, aspirin was added as there has been data showing lower frequency and severity of stroke‐like episodes in SWS patients, but this remains controversial [13]. CT venography is particularly useful for identifying the calcifications and vascular abnormalities associated with SWS, as seen in this patient [10]. The partially empty sella and prominent optic nerve sheaths may indicate increased intracranial pressure, a known complication of SWS [7]. These imaging findings, combined with the clinical presentation, provide strong evidence for the diagnosis of SWS, even in the absence of a visible port‐wine stain.

The underdiagnosis of SWS in African American patients is a significant concern, as the characteristic facial capillary malformations may be less apparent in darker skin tones. Port‐wine stains, which are present in approximately 90% of SWS cases, are often more challenging to detect in individuals with higher levels of melanin [10]. This diagnostic challenge may contribute to delayed or missed diagnoses in African American patients, particularly in cases where the cutaneous findings are subtle or absent. In this patient, the absence of a previously reported port‐wine stain may reflect either the difficulty of detecting the lesion in darker skin or the possibility of a “*forme fruste”* presentation of SWS, in which cutaneous features are minimal or absent [4]. This highlights the need for increased awareness of the variability in SWS presentations across different skin types and the importance of relying on neuroimaging and clinical findings to confirm the diagnosis. In our patient, her facial “birthmark” was not connected previously to her migraine or childhood seizure history, which could have prompted further workup.

The management of SWS is multidisciplinary, involving neurologists, ophthalmologists, and dermatologists. Antiepileptic drugs (AEDs) are the mainstay of treatment for seizures, but refractory cases may require surgical intervention, such as focal cortical resection or hemispherectomy [9]. In this case, the patient’s status epilepticus necessitated aggressive treatment with intravenous AEDs and continuous EEG monitoring. Emerging therapies, such as mTOR inhibitors, are being investigated for their potential to modulate abnormal vascular proliferation in SWS [14]. Low‐dose aspirin is often recommended to reduce the risk of stroke‐like episodes, although its efficacy in SWS remains controversial [13]. The elevated lactate level in this patient suggests tissue hypoxia, which may have contributed to the acute infarct. Addressing secondary complications, such as glaucoma and cognitive impairment, is also critical in the long‐term management of SWS [7].

Recent advances in research have focused on the role of the GNAQ mutation in the pathogenesis of SWS and its potential as a therapeutic target [2]. Preclinical models have demonstrated that mTOR pathway inhibition can reduce vascular malformations and improve neurological outcomes [14]. Additionally, advances in neuroimaging, such as quantitative MRI and PET, have improved the ability to monitor disease progression and treatment response in SWS [8]. These developments offer hope for more targeted and effective treatments for SWS, particularly in adult patients with progressive or refractory symptoms.

## 4. Conclusion

This case highlights the diagnostic and management challenges of SWS in adulthood, particularly in African American patients, where the characteristic cutaneous features may be less apparent. The patient’s presentation with status epilepticus and acute infarct underscores the fluctuating or episodic neurologic nature of SWS and the need for prompt diagnosis and management. Advances in understanding the molecular pathogenesis of SWS, particularly the role of the GNAQ mutation, offer hope for targeted therapies in the future. Multidisciplinary care remains essential to address the diverse neurological, ophthalmological, and dermatological manifestations of this complex disorder. Further research is needed to optimize treatment strategies and improve outcomes for patients with SWS, particularly in underrepresented populations where underdiagnosis may be more common.

## Funding

No funding was received for this manuscript.

## Consent

The patient provided informed written consent for publication of this case.

## Conflicts of Interest

The authors declare no conflicts of interest.

## Data Availability

Data sharing is not applicable to this article as no datasets were generated or analyzed during the current study.

## References

[1] Adams M. E. , Aylett S. E. , Squier W. , and Chong W. , A Spectrum of Unusual Neuroimaging Findings in Patients with Suspected Sturge-Weber syndrome, AJNR American Journal of Neuroradiology. (2009) 30, no. 2, 276–281, 10.3174/ajnr.A1350, 2-s2.0-60049083039. 19050205 PMC7051391

[2] Shirley M. D. , Tang H. , Gallione C. J. , Baugher J. D. , Frelin L. P. , Cohen B. , North P. E. , Marchuk D. A. , Comi A. M. , and Pevsner J. , Sturge–Weber syndrome and port-wine Stains Caused by Somatic Mutation in GNAQ, New England Journal of Medicine. (2013) 368, no. 21, 1971–1979, 10.1056/NEJMoa1213507, 2-s2.0-84877957142. 23656586 PMC3749068

[3] Cousyn L. , Leclercq D. , Ta M. C. et al., Late-Onset Status Epilepticus Associated with Isolated Leptomeningeal Angioma and Sturge–Weber syndrome–related GNA11 Pathogenic Variation, Neurology. (2023) 101, no. 22, 1021–1022, 10.1212/WNL.0000000000207839. 37813580 PMC10727224

[4] Hildebrand M. S. , Harvey A. S. , Malone S. et al., Somatic GNAQ Mutation in the Forme Fruste of Sturge–Weber syndrome, Neurology: Genetics. (2018) 4, no. 3, 10.1212/NXG.0000000000000236, 2-s2.0-85053879930. PMC5931068 29725622

[5] Rim J. , Santucci J. , Yan C. , and Morren J. , Late Presentation of Seizures in a 54-year-old Male with Sturge–Weber syndrome, Neurology. (2020) 94, no. 15 Supplement, 10.1212/WNL.94.15_supplement.329.

[6] Zecchin D. , Knöpfel N. , Gluck A. K. et al., GNAQ/GNA11 Mosaicism Causes Aberrant Calcium Signaling Susceptible to Targeted Therapeutics, Journal of Investigative Dermatology. (2024) 144, no. 4, 811–819, 10.1016/j.jid.2023.08.028. 37802293 PMC10957341

[7] Sabeti S. , Dobyns W. B. , Keating R. F. , Bitrian E. , Blieden L. S. , Brandt J. D. , Burkhart C. , Chugani H. T. , Falchek S. J. , Jain B. G. , Juhasz C. , Loeb J. A. , Luat A. , Pinto A. , Segal E. , Salvin J. , and Kelly K. M. , Consensus Statement for the Management and Treatment of Sturge-Weber syndrome: Neurology, Neuroimaging, and Ophthalmology Recommendations, Pediatric Neurology. (2021) 121, 59–66, 10.1016/j.pediatrneurol.2021.04.013. 34153815 PMC9107097

[8] Juhász C. , Batista C. E. A. , Chugani D. C. , Muzik O. , and Chugani H. T. , Evolution of Cortical Metabolic Abnormalities and Their Clinical Correlates in Sturge-Weber syndrome, European Journal of Paediatric Neurology. (2007) 11, no. 5, 277–284, 10.1016/j.ejpn.2007.02.001, 2-s2.0-34547576421. 17408998 PMC2020508

[9] Kossoff E. H. , Buck C. , and Freeman J. M. , Outcomes of 32 Hemispherectomies for Sturge-Weber syndrome Worldwide, Neurology. (2002) 59, no. 11, 1735–1738, 10.1212/01.WNL.0000035639.54567.5C, 2-s2.0-0037058798. 12473761

[10] Ramirez E. L. and Jülich K. , Sturge–Weber syndrome: An Overview of History, Genetics, Clinical Manifestations, and Management, Seminars in Pediatric Neurology. (2024) 51, 10.1016/j.spen.2024.101151. 39389653

[11] Juhász C. , Toward a Better Understanding of Stroke-like Episodes in Sturge–Weber syndrome, European Journal of Paediatric Neurology. (2020) 25, 3–10, 10.1016/j.ejpn.2020.02.005. 32107105 PMC7822069

[12] Yeom S. and Comi A. M. , Updates on Sturge–Weber syndrome, Stroke. (2022) 53, no. 12, 3769–3779, 10.1161/STROKEAHA.122.038585. 36263782 PMC11062639

[13] Lance E. I. , Sreenivasan A. K. , Zabel T. A. , Kossoff E. H. , and Comi A. M. , Aspirin Use in Sturge-Weber syndrome: Side Effects and Clinical Outcomes, Journal of Child Neurology. (2013) 28, no. 2, 213–218, 10.1177/0883073812463607, 2-s2.0-84872837516. 23112247 PMC4373084

[14] Sebold A. J. , Day A. M. , Ewen J. et al., Sirolimus Treatment in Sturge-Weber syndrome, Pediatric Neurology. (2021) 115, 29–40, 10.1016/j.pediatrneurol.2020.10.013. 33316689 PMC8209677

